# Signature of antiphase boundaries in iron oxide nanoparticles

**DOI:** 10.1107/S1600576721010128

**Published:** 2021-11-16

**Authors:** Tobias Köhler, Artem Feoktystov, Oleg Petracic, Nileena Nandakumaran, Antonio Cervellino, Thomas Brückel

**Affiliations:** a Jülich Centre for Neutron Science JCNS at Heinz Maier-Leibnitz Zentrum MLZ, Forschungszentrum Jülich GmbH, 85748 Garching, Germany; b Jülich Centre for Neutron Science JCNS-2 and Peter Grünberg Institute PGI-4, JARA-FIT, Forschungszentrum Jülich GmbH, 52425 Jülich, Germany; c Lehrstuhl für Experimentalphysik IV C, RWTH Aachen University, 52056 Aachen, Germany; d Swiss Light Source, Paul Scherrer Institut, 5232 Villigen PSI, Switzerland

**Keywords:** nanoparticles, X-ray powder diffraction, Monte Carlo simulations, Debye scattering equation, antiphase boundaries

## Abstract

Iron oxide nanoparticles find a wide variety of applications, including targeted drug delivery and hyperthermia in advanced cancer treatment methods. An important property of these particles is their maximum net magnetization, which has been repeatedly reported to be drastically lower than the bulk reference value. Previous studies have shown that planar lattice defects known as antiphase boundaries (APBs) have an important influence on the particle magnetization. The influence of APBs on the atomic spin structure of nanoparticles with the γ-Fe_2_O_3_ composition is examined via Monte Carlo simulations, explicitly considering dipole–dipole interactions between the magnetic moments that have previously only been approximated. For a single APB passing through the particle centre, a reduction in the magnetization of 3.9% (for 9 nm particles) to 7.9% (for 5 nm particles) is found in saturation fields of 1.5 T compared with a particle without this defect. Additionally, on the basis of Debye scattering equation simulations, the influence of APBs on X-ray powder diffraction patterns is shown. The Fourier transform of the APB peak profile is developed to be used in a whole powder pattern modelling approach to determine the presence of APBs and quantify them by fits to powder diffraction patterns. This is demonstrated on experimental data, where it could be shown that the number of APBs is related to the observed reduction in magnetization.

## Abstract 2

Antiphase boundaries (APBs) in iron oxide nanoparticles are studied by means of simulations. A strong influence on the spin structure is determined with Monte Carlo simulations, while *hkl*-dependent peak broadening useful for identifying APBs in real samples is found from Debye scattering equation simulations.

## 1. Introduction

The increasing number of existing and potential applications for superparamagnetic iron oxide nanoparticles (SPIONs) has sparked considerable interest from both a technological and a scientific point of view. For example, seals and adaptive dampers make use of ferrofluids containing SPIONs (Bailey, 1983 ▸; Raj *et al.*, 1995 ▸; Sun, 2002 ▸). Due to their biocompatibility, SPIONs are also used in the field of medicine for tissue repair (Bock *et al.*, 2010 ▸; Pareta *et al.*, 2008 ▸; Tran & Webster, 2009 ▸) and targeted drug delivery (Dobson, 2006 ▸; Veiseh *et al.*, 2010 ▸; Sun *et al.*, 2008 ▸), as contrast agents for magnetic imaging (Josephson *et al.*, 1999 ▸; Semelka & Helmberger, 2001 ▸; Corot *et al.*, 2006 ▸; Jun *et al.*, 2008 ▸; Laurent *et al.*, 2008 ▸; Sun *et al.*, 2008 ▸; Ma *et al.*, 2015 ▸) and magnetic particle imaging (Gleich & Weizenecker, 2005 ▸), and in cancer treatment by magnetic hyperthermia (Johannsen *et al.*, 2005 ▸; Hergt *et al.*, 2006 ▸; Gonzales-Weimuller *et al.*, 2009 ▸; Krishnan, 2010 ▸; Kumar & Mohammad, 2011 ▸; Laurent *et al.*, 2011 ▸; Deatsch & Evans, 2014 ▸). The structural and magnetic properties of SPIONs have been subject to extensive research, for example on the origin of the reduction of the saturation magnetization in γ-Fe_2_O_3_ and Fe_3_O_4_ nanoparticles depending on the particle size (Nedel­koski *et al.*, 2017 ▸; Sharifi Dehsari *et al.*, 2018 ▸; Disch *et al.*, 2012 ▸; Nemati *et al.*, 2018 ▸; Herlitschke *et al.*, 2016 ▸; Wetterskog *et al.*, 2013 ▸; Andersen *et al.*, 2021 ▸). The origin of this reduction appears to be a combination of several effects, such as spin canting near defects (Disch *et al.*, 2012 ▸; Herlitschke *et al.*, 2016 ▸), surface spin canting and the presence of antiphase boundaries (APBs) (Nedelkoski *et al.*, 2017 ▸; Köhler *et al.*, 2021 ▸). The last have a strong influence on the magnetic properties of iron oxide nanoparticles (Köhler *et al.*, 2021 ▸) and are the subject of this study.

Antiphase boundaries are interfaces in a crystalline structure that separate domains of the same ordered phase which are displaced relative to each other (Kikuchi & Cahn, 1979 ▸; Allen & Cahn, 1979 ▸). They occur especially in metallic systems with disorder–order transitions, *e.g.* the extensively studied alloy Cu_3_Au (Wilson, 1943 ▸; Fisher & Marcinkowski, 1961 ▸; Wilson & Zsoldos, 1966 ▸; Warren, 1990 ▸; Scardi & Leoni, 2005 ▸). APBs in Fe_3_O_4_ thin films have also been the subject of numerous investigations (Hibma *et al.*, 1999 ▸; Eerenstein *et al.*, 2001 ▸; Eerenstein, Palstra, Hibma & Celotto, 2002 ▸; Eerenstein, Palstra, Saxena & Hibma, 2002 ▸; Luysberg *et al.*, 2009 ▸; Gilks *et al.*, 2013 ▸). Recent work has shown that APBs are stable in iron oxide nanoparticles and are, to a large extent, responsible for specific magnetic properties (Wetterskog *et al.*, 2013 ▸; Nedelkoski *et al.*, 2017 ▸; Köhler *et al.*, 2021 ▸). APBs in iron oxide nanoparticles are assumed to originate from oxidation processes during the synthesis of these particles, where the initial FeO transforms into Fe_3_O_4_ and γ-Fe_2_O_3_. As observed from dark-field transmission electron microscopy, the nucleation of the spinel phase Fe_3_O_4_ occurs at multiple spots in the particle. When the various subdomains eventually meet with continuing oxidation of the particle, the respective structures of the domains might be shifted, resulting in an APB. Upon further oxidation Fe_3_O_4_ is transformed into the structurally very similar γ-Fe_2_O_3_, while the previously created APBs persist (Wetterskog *et al.*, 2013 ▸).

First-principles calculations showed that stable APBs form on the {110} planes of Fe_3_O_4_ thin films (McKenna *et al.*, 2014 ▸). The two most stable configurations are crystal translations of 1/4*a*[110] (APB-I) and 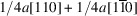 (APB-II). APB-I has a low formation energy of 102 mJ m^−2^ because the only distortion resulting from this shift is found between octahedral and tetrahedral Fe sites at the interface. The oxygen sublattice is not affected, and the total number of tetrahedral and octahedral sites remains the same. APB-II has a calculated formation energy of 954 mJ m^−2^, associated with a larger degree of structural distortion induced by the lattice translation. The most notable feature of APB-I is the breaking of the long *B*-site chains perpendicular to the boundary plane, which results in a modification of the bond angle between iron atoms in these chains from 90 to 180° and a subsequent change of the magnetic superexchange interaction from ferromagnetic to antiferromagnetic. As noted before, this has an impact on the magnetic properties of iron oxide nanoparticles (Nedel­koski *et al.*, 2017 ▸) and thin films (Moreno *et al.*, 2021 ▸). The resulting antiferromagnetic superexchange between octa­hedral Fe^3+^ ions across the boundary has been confirmed for Fe_3_O_4_ thin films via magneto-resistance measurements (Eerenstein *et al.*, 2001 ▸). Simulations of the effect of APBs on the atomic spin structure in Fe_3_O_4_ nanoparticles (10 nm) in fields of 5 T suggested a tilting of the net magnetization for the two structural sub­domains separated by the APB away from the applied field direction, thus resulting in magnetic domains leading to the observed reduced total magnetization (Nedelkoski *et al.*, 2017 ▸). However, in simulations of structures containing this kind of defect, dipole–dipole interactions are often neglected, or only approximated by a macrocell method, due to the high computational effort required to calculate the dipole–dipole energy term that contains all interatomic distances (Nedel­koski *et al.*, 2017 ▸; Evans *et al.*, 2014 ▸). Nevertheless, the long-range nature of this interaction might produce non-negligible effects on the atomic spin structure that could have been overlooked by the previous approximations. Previous studies also rely on high-resolution transmission electron microscopy techniques for the detection of APBs in iron oxide nanoparticles (Wetterskog *et al.*, 2013 ▸; Nedelkoski *et al.*, 2017 ▸).

In this work we employ atomistic Monte Carlo simulations explicitly considering the dipole–dipole interactions to study the effect of APBs on the magnetic spin structure of iron oxide nanoparticles. Furthermore, we show via simulations of X-ray powder diffraction patterns and by comparison with experimental data that APBs in nanoparticles produce a distinct signature in the diffraction profile that concerns the widths of diffraction peaks, which can be used to check for the presence of APBs and to quantify them.

## 2. Methods

### 2.1. Computer simulations

In order to study the effect of APBs on the spin structure and the crystal structure, simulations were performed. Spin structures were simulated with an atomistic Monte Carlo approach utilizing the Metropolis algorithm (Newman & Barkema, 1999 ▸). The influence of APBs on powder diffraction patterns was simulated with the Debye scattering equation (Debye, 1915 ▸). Since for small crystals γ-Fe_2_O_3_ appears to be the more common composition (Demortiere *et al.*, 2011 ▸; Andersen *et al.*, 2021 ▸) we focus on this compound. The unit cell contains eight tetrahedrally coordinated (*A* site) iron sites and 16 octahedral (*B* site) iron sites in addition to 32 oxygen atoms. Vacancies are distributed on *B* sites in order to achieve charge neutrality (Andersen *et al.*, 2021 ▸). The vacancies have been observed to order, thus reducing the unit-cell symmetry from cubic to tetragonal (Shmakov *et al.*, 1995 ▸; Greaves, 1983 ▸; Jørgensen *et al.*, 2007 ▸). For the sake of reducing the number of parameters in this study, random vacancy distribution is assumed on the octahedral sites, yielding the cubic space group 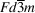 . The magnetic structure of γ-Fe_2_O_3_ can be described by octahedral and tetrahedral sublattices where the atomic magnetic moments are antiferromagnetically aligned via superexchange interactions between Fe^3+^ ions on *A* and *B* sites. This results in ferrimagnetic ordering of the magnetic moments in the unit cell (Lee & Lee, 2006 ▸; Andersen *et al.*, 2021 ▸). A net magnetic moment per unit cell can be observed due to the larger number of Fe ions on *B* sites than on *A* sites, resulting in a bulk saturation magnetization of 82 A m^2^ kg^−1^ at 0 K (Coey, 2010 ▸).

We emphasize that the nanoparticles considered for the simulations are idealized in the sense that no spatial gradients in the iron or oxygen concentrations or in the lattice parameters are included. Moreover, no surface anisotropy effects resulting from facets that might be present in real samples were considered. Previous studies have found that, while surface effects, such as a non-magnetic surface, have an influence on the magnetic properties of iron oxide nanoparticles, they alone are not able to explain the reduced magnetization of these particles (Disch *et al.*, 2012 ▸; Köhler *et al.*, 2021 ▸). In the simulations presented here we do not consider them, since these surfaces are not expected to alter the effects of APBs. For real particles, however, a non-magnetic surface might contribute to the observed net magnetization.

#### 2.1.1. Monte Carlo simulations

The energy for a semi-classical Heisenberg spin model of magnetic moment vectors in γ-Fe_2_O_3_ nanoparticles used in this work is given by 

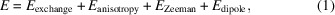

where the contributions are due to the magnetic exchange interactions of nearest neighbours, the cubic anisotropy, the applied field through the Zeeman interaction and the magnetic dipole–dipole interactions of atomic spins.

The strongest contribution is the exchange energy, and this is given by 

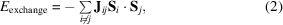

where the sum runs over the nearest neighbours, **S**_*i*_ and **S**_*j*_ are the spin vectors located at the atom positions *i* and *j*, and **J**_*ij*_ is the relevant exchange constant. The numerical values used for the inter-sublattice (*AB*) and intra-sublattice (*AA* and *BB*) exchange constants are *J*_*AA*_/*k*_B_ = −21.0 K, *J*_*BB*_/*k*_B_ = −8.6 K and *J*_*AB*_/*k*_B_ = −28.1 K, respectively, where *k*_B_ is the Boltzmann constant in joules per kelvin (Kodama & Berkowitz, 1999 ▸). For the modified superexchange interaction across the APB, *J*_*BB*_/*k*_B_ is taken as 103.3 K if both octahedral iron atoms are placed at the APB (Nedelkoski *et al.*, 2017 ▸).

The magnetocrystalline anisotropy contribution is given by (Evans *et al.*, 2014 ▸) 

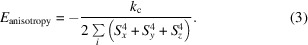

A cubic magnetocrystalline anisotropy constant *k*_c_ of 3.2 × 10^−25^ J per atom was used (Nedelkoski *et al.*, 2017 ▸). *S*_*x*_, *S*_*y*_ and *S*_*z*_ are the vector components of the classical spin vector **S**. Surface anisotropy was not explicitly considered, but surface canting of spins might still be visible in the simulations, due to the reduced number of neighbours and the rough surface introduced by the spherical cutting of the particle.

The interaction of the particle with the external magnetic field is described by the Zeeman energy (Blundell, 2001 ▸), 

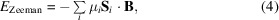

where **B** is the applied field in tesla and μ_*i*_ is the magnetic moment of atom *i* taken as 5 μ_B_.

The energy contribution from magnetic dipole interactions is 

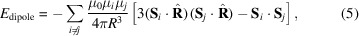

where 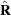 and *R* are the distance vector and the distance between atoms *i* and *j*, respectively. Due to the long-range nature of these interactions the sum has to run over all Fe ions in the particle, resulting in a computational burden that scales with *R* ^3^. In this work the dipole–dipole interactions are calculated for all spins directly without further approximations.

Only the ground states are considered here, *i.e.* the temperature entering the Boltzmann distribution of the Metropolis algorithm is set to a value of 0.01 K. For the spin structure simulations, a field of 5 T was applied and 20 starting configurations of the particle with random placements of the vacancies were averaged after allowing equilibration of the system with 5000 Monte Carlo steps, *i.e.* every spin in the structure has statistically been moved 5000 times. For the hysteresis simulations, 8000 steps were used per field step of 0.1 T and 20 different configurations in 20 orientations, meaning a total of 400 simulated loops were averaged.

**Figure 1:**
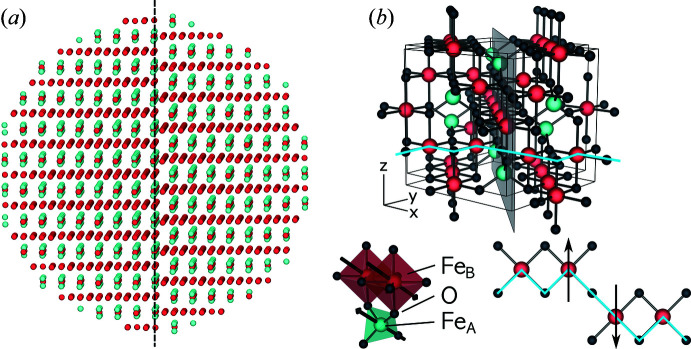
(*a*) The spherical nanoparticle structure used for the Monte Carlo and Debye scattering equation simulations (visualized with *MayaVi*; Ramachandran & Varoquaux, 2011 ▸). Oxygen atoms have been omitted for better visibility of the iron atoms. The structure contains an APB through the particle centre (black dashed line). Pale-green dots correspond to iron atoms on tetrahedrally coordinated *A* sites. Red dots are the *B*-site iron atoms that are octahedrally coordinated by oxygen atoms [black dots in panel (*b*)]. Vacancies are placed randomly on *B* sites. (*b*) The 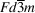 unit cell of the γ-Fe_2_O_3_ structure [generated with *Vesta* (Momma & Izumi, 2011 ▸)]. The grey plane indicates the shifting plane of the 1/4*a*[110] APB. The outlines of two unit cells are also shown in black. The blue line highlights a *B*-site chain perpendicular to the APB, where the translation results in a change of bond angles from 90° to 180° across the boundary and subsequent antiparallel alignment of the atomic spins (drawn below in top view).

The particle is built up by repeating the 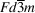 γ-Fe_2_O_3_ unit cell *n* times in all space directions and removing all atoms outside a bounding sphere with a diameter of *D* = 5.0, 6.7 or 9.2 nm. Oxygen atoms were considered as non-magnetic and thus were not included in the structural model for Monte Carlo simulations. Random vacancies were introduced at the octahedral lattice sites, giving an average occupation of 0.88%. This is the theoretical occupation of this site in bulk maghemite. Note, however, that site occupancies for real samples might vary (Grau-Crespo *et al.*, 2010 ▸; Cervellino *et al.*, 2014 ▸; Andersen *et al.*, 2021 ▸). The APB was introduced into the nanoparticle by shifting one half of the crystal structure by 1/4*a*[110], resulting in the configuration shown in Fig. 1 ▸, where the APB is placed at the particle centre and assumed to be planar. The last assumption is based on energetic considerations, where a minimization of interfacial energies is expected to lead to planar APBs (Bragg, 1940 ▸; Allen & Cahn, 1979 ▸). The placement of the APB at the particle centre is based on experimental observations using high-resolution transmission electron microscopy (Levy *et al.*, 2011 ▸; Nedelkoski *et al.*, 2017 ▸; Köhler *et al.*, 2021 ▸).

#### 2.1.2. Debye scattering equation

The Debye scattering equation (DSE) is given by 

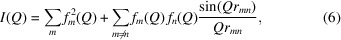

where *f*_*n*_(*Q*) is the atomic scattering or form factor of atom *n*, and *r*_*mn*_ is the distance between atoms at *r*_*m*_ and *r*_*n*_. The scattering vector magnitude is defined as 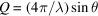 . Derivations can be found in the original work by Peter Debye (1915 ▸) or in the work of Farrow & Billinge (2009 ▸). Thermal vibrations of atoms were not considered, since they produce only a decrease in peak intensity for increasing scattering angles and a diffuse background, and thus are not expected to affect substantially the peak shapes and widths in the considered *Q* range (Warren, 1990 ▸). Due to the spherical average used in the derivation of the DSE, the calculated pattern corresponds to the particle in all possible orientations. Since only the pair-wise atomic distances *r*_*mn*_ enter equation (6) the input structure is not required to be periodic, which allows the study of effects that alter the periodicity of crystal structures such as APBs. *I*(*Q*) in equation (6) is the average coherent scattering intensity.

The first part of equation (6) is the self-scattering contribution from an atom with itself, *i.e.* *m* = *n* and hence *r*_*mn*_ = 0, and is equal to the number of atoms *N* times the average of the squared atomic form factors 〈*f*(*Q*)^2^〉. The atomic form factors are approximated by the interpolation 

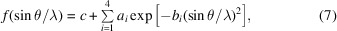

where *a*, *b* and *c* are the Cromer–Mann coefficients as given in *International Tables for Crystallography* (Cromer & Mann, 1968 ▸; Brown *et al.*, 2006 ▸). This approximation is reliable up to *Q* = 25 Å^−1^, enough for the scattering range considered in this work. The nanoparticle structures used for the DSE are set up in the same way as for the Monte Carlo simulations, but here oxygen atoms are of course considered as part of the structure.

### 2.2. X-ray powder diffraction

Synchrotron X-ray powder diffraction experiments were performed on beamline MS-X04SA of the Swiss Light Source at the Paul Scherrer Institut (Villigen, Switzerland) (Willmott *et al.*, 2013 ▸) on a dried sample of 15.6 nm nanoparticles with oleic acid coating obtained from Ocean NanoTech LLC (San Diego, California, USA). The instrumental contribution was determined by measurements of the NIST standard 660a LaB_6_. Measurements were performed with an X-ray wavelength of 0.4329 Å. The nanoparticle sample was filled into a glass capillary with 0.9 mm interior diameter, which was inserted into a brass fitting and loaded into the sample cassette. For the measurement the sample was picked up by the sample-changer robot and inserted horizontally in the sample space. During the measurement the capillary was rotated around its long axis. After collection of the data with a 1D detector the instrument-related corrections were applied immediately. The measured data were corrected for the empty capillary and the empty beam contributions.

## 3. Results and discussion

### 3.1. Influence of APBs on the atomic spin structure

**Figure 2:**
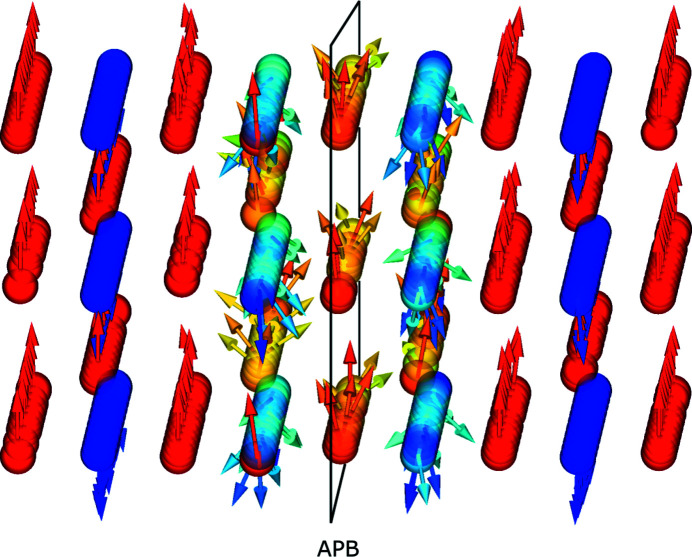
An enlargement of the spin structures of the 9.2 nm particles with an APB at the centre, indicated with a black rectangle. The structures were simulated under consideration of dipole–dipole interactions (visualized with *MayaVi*). A field of 5 T was applied along the vertical direction. Full parallel (octahedral sites) and antiparallel (tetrahedral sites) alignment with the applied field is shown with red and blue colours, respectively. Intermediate colours indicate spin canting away from perfect alignment. The introduction of an APB leads to a disturbance in the spin structure due to the antiferromagnetic exchange interaction across the boundary.

The strong antiferromagnetic superexchange across the APB leads to spin canting of the involved cations that persists even in high fields of 5 T (Fig. 2 ▸). From a visual inspection of the spin arrangement, the spin canting is confined to the region directly next to the APB. The dipole–dipole inter­actions have only a small influence on this spin arrangement (see supplementary Fig. S5) and also on the averaged net magnetization. This may be expected, since the spin structure on this local scale is mainly influenced by the much stronger exchange interactions. Additionally, shape anisotropy is expected to be negligible for the considered spherical particle shapes. However, for non-spherical particles the resulting shape anisotropy should be taken into account.

**Figure 3:**
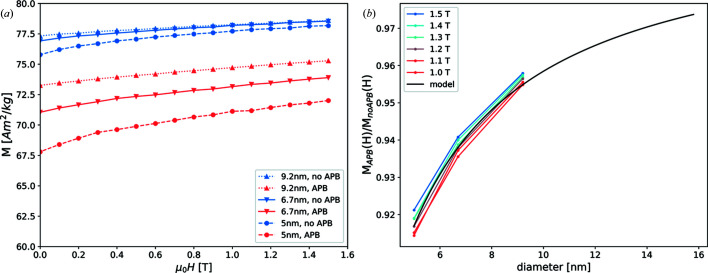
(*a*) Simulated magnetization versus applied field data for 5.0, 6.7 and 9.2 nm particles. Each curve is the result of an average of 20 different particle configurations in 20 orientations each. 8000 Monte Carlo steps were used for each field step of 0.1 T. (*b*) Magnetization ratios of curves obtained for particles with APBs and those without, shown as a function of the particle diameter for different applied fields. The black line corresponds to an approximation by equation (8) with *t* = 0.33 nm.

The macroscopic magnetization is strongly reduced for all considered particle sizes [Fig. 3 ▸(*a*)]. For the largest considered particles the magnetization is reduced by 3.9% in a field of 1.5 T. This reduction becomes slightly stronger in a weaker field, 4.3% at 0.9 T. The magnetization of the smaller particles exhibits a similar field dependence. The overall relative reduction of the net magnetization compared with the simulated particles with no APBs gets stronger with decreasing particle size [Fig. 3 ▸(*b*)]. This size dependence reflects the relative proportion of atomic moments directly influenced by APBs. Due to the planar nature of this type of defect, the relative volume immediately affected decreases with increasing particle size. For the case of a single APB the reduction in magnetization can be approximated by 

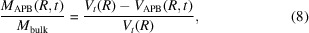

where *M*_APB_ is the magnetization of a particle containing an APB and *M*_bulk_ is the bulk saturation magnetization that is expected for a particle without any defects. The spin canting at the APB leading to the reduced magnetization can be approximated by assuming a cylindrical slab within the particle, where the net magnetization is zero, according to *V*_APB_ = π*R* ^2^ *t*, where *t* denotes the thickness of this slab. In general, *t* is a function of the applied field. In Fig. 3 ▸(*b*) the black line corresponds to an approximation by equation (8) with *t* = 0.33 nm.

A comparison with experimental data shows that perfectly crystalline particles can exhibit almost bulk-like saturation magnetization (Herynek *et al.*, 2021 ▸) while particles containing APBs have been found to show strongly reduced magnetization (Köhler *et al.*, 2021 ▸). In the latter case a reduction of about 13% for 15.6 nm particles was attributed to APBs in an applied field of 1.0 T. A similar reduction in saturation magnetization was also observed for larger cubic particles, where evidence for the presence of APBs was found (Wetterskog *et al.*, 2013 ▸). These reductions are stronger than what we found for 9.2 nm particles in comparable fields. As mentioned above, the larger particle size may allow for the presence of more than one APB. This possibility is explored in the next section for the mentioned 15.6 nm particles. Additionally, it is likely that the APBs in real samples are not as perfect on the atomic level as was assumed in our simulations. These local lattice distortions might contribute to the stronger influence on the net magnetization. Finally, the absolute reduction of the net magnetization also depends on the value chosen for the antiferromagnetic superexchange constant, which has only been extrapolated and not yet experimentally confirmed (Nedelkoski *et al.*, 2017 ▸).

**Figure 4:**
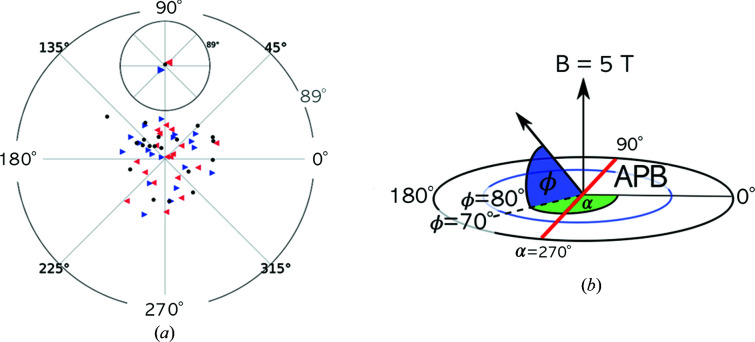
(*a*) Net magnetization vectors for particle halves to the left (left-facing triangles) and to the right (right-facing triangles) of the APB, simulated with consideration of dipole–dipole interactions. The total net magnetization vectors are drawn as black dots. (*b*) A sketch illustrating the construction of the polar plots.

As mentioned in the *Introduction* [Sec sec1], magnetic domain formation in particles expected to be superparamagnetic has been proposed to explain the observed reduced magnetization (Nedelkoski *et al.*, 2017 ▸). In Fig. 4 ▸(*a*) the net magnetizations of the particle halves on either side of the APB of the simulated particle structures are shown in a polar representation. Here, the normalized magnetization vectors of the simulated structures are placed at the origin. A projection plane is chosen perpendicular to the applied field direction such that fully aligned vectors that are either parallel or antiparallel to the field vector correspond to points at the centre of the plot. The position of the projected points is defined by an azimuthal angle (α) and an elevation angle (ϕ), where the former is drawn on the perimeter and the latter gives the radial distance from the centre. The elevation angle is defined as the angle between the spin vector and the plane perpendicular to the field vector, thus resulting in an angle of ±90° for full alignment. For the plots the absolute values of the elevation angles are used. The azimuthal angle defines the orientation of the spin vector around the field vector. The APB is positioned on the line connecting azimuthal angles 90 and 270° [Fig. 4 ▸(*b*), red line]. Left- and right-facing triangles correspond to the left and right halves, respectively. Black dots show the total magnetization vector of the particles. The averaged net magnetization vectors for the particle halves are tilted with respect to the applied field, as shown in the inset of Fig. 4 ▸(*a*). These results seem to indicate that there is a collective canting of spins on either side of the APB, in agreement with previous simulations by Nedelkoski *et al* (2017 ▸)

**Figure 5:**
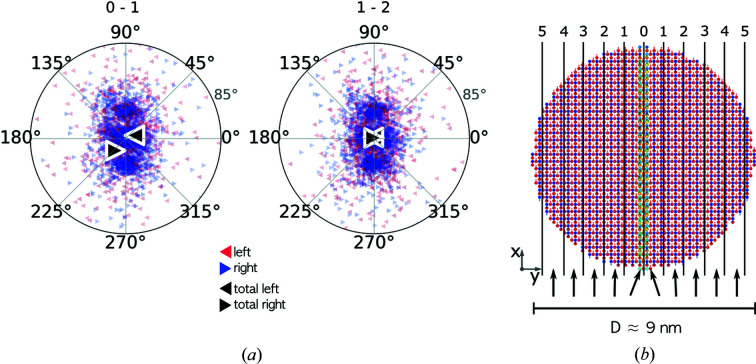
(*a*) Net magnetization vectors for slices to the left- and to the right-hand side of the APB (large black triangles) together with the individual spin vectors (small triangles) for 9.2 nm particles. (*b*) A sketch of a particle with *D* ≃ 9 nm, showing the numbered slices. The slab positions indicated at the top of panel (*a*) correspond to the slice numbers shown in (*b*). The canting of spins is confined to the region within one unit cell around the APB, and this is indicated in an exaggerated way by the arrows below the particle shown in panel (*b*).

However, an inspection of the net magnetization in slabs at different distances from the APB shows that this strong tilting mainly affects the region within one unit-cell distance of the APB. For the slabs farther away the canting is strongly reduced. This effect is sketched in a highly exaggerated manner in Fig. 5 ▸(*b*), with the arrows symbolizing the net magnetization direction of the slabs. This shows that the particles remain in the single-domain state, as expected from energetic considerations, and no multi-domain state forms. This is also in agreement with neutron scattering experiments that should be able to detect a multi-domain state (Bedanta *et al.*, 2015 ▸; Disch *et al.*, 2012 ▸; Köhler *et al.*, 2021 ▸). The direct calculation of dipole–dipole interactions has only a small influence on this behaviour (supplementary Figs. S7 and S8). For smaller particles, a gradual realignment with increasing distance from the APB can also be observed, where significant canting of moments is only observed within one unit-cell distance of the APB (supplementary Fig. S6).

### 3.2. Influence of APBs on X-ray powder diffraction patterns

The X-ray powder diffraction line profile is affected by several contributions, including the crystallite shape and size, lattice strains, dislocations, twin and stacking faults, grain surface relaxations, compositional fluctuations, and APBs. In the whole powder pattern modelling (WPPM) approach introduced by Scardi *et al.* (2010 ▸) these different contributions can be deconvoluted by using the Fourier transforms of the profiles related to the individual effects. In this approach the peak profile of a powder diffraction peak for a spherical nanoparticle is obtained via 

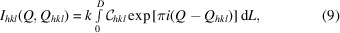

where *Q*_*hkl*_ is the peak position, *D* is the particle diameter, *L* is the distance in real space along [*hkl*] and 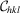 denotes the product of all Fourier transforms of the profile contributions. The parameter *k* contains constant terms that are not explicitly considered here, including the absolute square of the structure factor |*F*_*hkl*_|^2^. In this work we focus primarily on the finite size and the APB contribution. For real samples the instrumental contribution might also need to be considered. Hence, in the present case 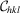 is given as 

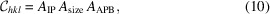

where *A*_IP_, *A*_size_ and *A*_APB_ are the Fourier coefficients related to the instrumental profile, the particle size and the APBs, respectively. Expressions for these components are shown in Appendices *A* [App appa]–*C* [App appb] [App appc]. To the best of our knowledge, the contribution *A*_APB_ for APBs in maghemite and magnetite has not been shown before and is developed in this work in Appendix *B* [App appb] using the theory presented by Wilson and Zsoldos (Wilson, 1943 ▸; Wilson & Zsoldos, 1966 ▸) and Warren (1990 ▸). Consideration of further line-broadening effects is straightforward by inclusion of the corresponding Fourier transforms of the resulting peak profiles in equations (9) and (10).

**Figure 6:**
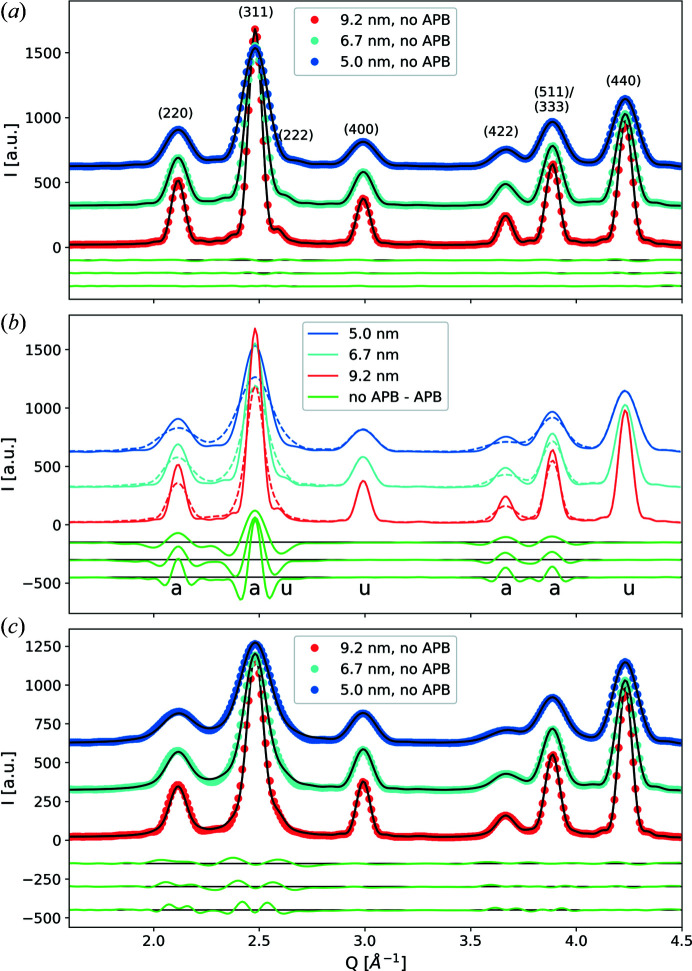
(*a*) X-ray powder pattern simulations using equation (6) for spherical particles with no APB with diameters of 5.0, 6.7 and 9.2 nm. The simulated data were normalized to the number of scatterers and offset for clarity. Each fitted curve is a superposition of peak profiles according to equation (9). Difference curves between the data and the fits are shown below in the same order as the diffraction patterns from top to bottom. The fitting parameters were the particle size and the peak intensities. (*b*) A comparison of patterns simulated for particles with and without an APB at the centre. Difference curves between patterns simulated for particles without and with an APB show that some peaks are not affected, marked with ‘u’, while others are broadened, marked with ‘a’. (*c*) Fits to the simulated patterns for particles containing an APB at the centre using equation (9). The only fitting parameter was the APB probability δ. Integral peak intensities and particle sizes were fixed from the fits shown in panel (*a*).

The effect of particle size on simulated powder diffraction patterns is shown in Fig. 6 ▸(*a*). A decrease in particle size leads to an increase in the peak widths that affects all diffraction peaks. Fits to these curves with equation (9) only using the size component *A*_size_ return the input parameters of the particle diameters with high precision. The peak positions were constrained to the theoretical values of the used unit cell. Peak intensities were left unconstrained for the fit. A linear background was used to account for the small-angle scattering contribution creeping under the Bragg reflections.

Simulations of powder patterns with nanoparticles containing an APB through the particle centre as depicted in Fig. 1 ▸(*a*) show a distinct *hkl* dependence of the peak broadening [dashed lines in Fig. 6 ▸(*b*)]. Comparing the curves with patterns obtained from particles with no APB reveals that certain peaks are not affected at all, namely peaks 222, 400 and 440, marked with the letter ‘u’ in Fig. 6 ▸(*b*). This *hkl* dependence and its association with APBs has been recognized before, *e.g.* in γ-Al_2_O_3_ (Rudolph *et al.*, 2019 ▸) with a similar structure, in a powder of γ-Fe_2_O_3_ (Nakajima *et al.*, 1987 ▸) and in iron oxide nanoparticles (Köhler *et al.*, 2021 ▸). As shown in Appendix *B* [App appb], for the peaks with pairs of indices whose sums are multiples of four, no phase shifts due to the APB occur and thus the peaks are not affected. The contribution of the APB to the powder diffraction line profile can, as mentioned above, be included in equation (9) by the use of the Fourier transform of the APB peak profile *A*_APB_ as developed in Appendix *B* [App appb].

By using equation (9) with *A*_size_ and *A*_APB_, the finite size broadening and the APB effect are correctly considered and the particle diameter *D* and the APB probability δ can be obtained directly from fits to the data. Application to the simulated powder patterns for particles with one APB at the centre is shown in Fig. 6 ▸(*c*), where good agreement is obtained between a pattern generated by a superposition of peak profiles according to equation (9) and the simulations. As shown by Warren (1990 ▸), the APB does not change the integrated intensity of the peaks, so for the fits the scale factors of the peaks were constrained to the values obtained from the fits to particles with no APBs. The only fitting parameter was therefore the APB probability δ. By definition, δ is the total probability of an APB occurring within the distance given by the lattice parameter *a*_0_ along each real-space direction. Thus multiplication by the number of unit cells, *i.e.* *D*/*a*_0_, yields the probability per particle. This value should be the same for each simulated structure since only one APB was introduced for each particle size. From the fits to the simulated data shown in Fig. 6 ▸(*c*), parameters δ of 0.075, 0.055 and 0.041 were obtained for particle sizes of 5.0, 6.7 and 9.2 nm, respectively, which give the APB probabilities per particle diameter as 0.442, 0.442 and 0.451, respectively. Since the APB is always parallel to one real-space direction, the total probability is δ = 2/3δ_*x*_, where δ_*x*_ denotes the probability along *x*. For a spherical particle, an APB on the (110) plane is at an angle of 45° to the *x* axis and thus the phase shift is only observed for a fraction of the particle cross section, while for some parts the X-rays ‘see’ a structurally coherent particle (see supplementary Fig. S2). This fraction is given by the ratio of the area of the APB projection onto the *yz* plane to the particle cross section, resulting in a factor of 1/2^1/2^ in δ_*x*_. With that, the number of APBs at the particle centre is 

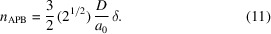

For the simulated patterns this yields 0.94 (1), 0.94 (1) and 0.96 (1) APBs with increasing particle size. The slight deviations from the expected value may be attributed to the imperfect spherical size of the smaller particles and to the fact that the description of the peak broadening due to APBs was developed under the assumption of large crystals. For a single off-centred APB, values for the number of APBs smaller than one are obtained by the use of equation (11), since the peak broadening is not as strong (see supplementary Fig. S1). This is related to a decrease in the ratio of the projected APB area to the sphere cross section.

### 3.3. Application to experimental data

**Figure 7:**
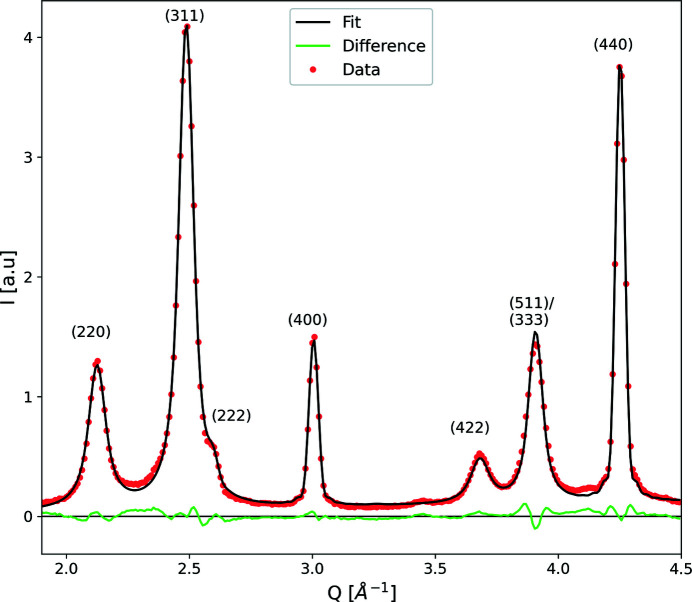
A profile fit to experimental data of iron oxide nanoparticles with diameters of 15.6 nm. Depicted are sections of a measurement over a wider *Q* range. The whole pattern is shown in supplementary Fig. S3. The fitted curve is a superposition of peak profiles according to equation (9). Fitting parameters were the particle size *D*, the APB probability δ, the peak intensities, the peak positions and a linear background. The difference between the data and the fit is shown with a green line.

A fit using equation (9) to experimental powder diffraction data obtained for nanoparticles with sizes of 15.6 nm is shown in Fig. 7 ▸. These particles were extensively characterized in a previous publication, where we found a significantly reduced magnetization compared with the bulk material of about 23% to 60 (1) A m^2^ per kg_FeOx_ (Köhler *et al.*, 2021 ▸). A magnetically dead surface layer of 0.3 (1) nm thickness was determined, but it was concluded that APBs are responsible for a drop in magnetization of about 13% compared with the bulk material.

The parameters for the fit shown in Fig. 7 ▸ were the peak positions, the peak intensities, the particle diameter *D*, the APB probability parameter δ and a linear background. Polydispersity was not considered in the fit and the instrumental contribution to the peak broadening was deemed insignificant compared with the finite size broadening (supplementary Fig. S4). Other sources of line broadening were also not considered. The fact that a good fit is obtained even with these approximations suggests that these contributions are not strong in the present sample. In particular, peaks 400 and 440 that are not affected by the APB would indicate if significant contributions of, for example, strain or dislocation broadening were present. However, the fact that a single particle size parameter produces a good fit to both peaks suggests that this is not the case. The particle diameter calculated from the fit is to 15.1 (2) nm, which is very close to the diameter of 15.6 (1) nm determined via small-angle X-ray scattering [see Köhler *et al.* (2021 ▸)]. A value of δ = 0.055 (3) was determined, resulting in 2.2 (1) APBs by the use of equation (11).

To estimate the impact of APBs on the magnetization, equation (8) with *t* = 0.33 nm, as determined from the Monte Carlo simulations, is used. The volume affected by the APB [*V*_APB_ in equation (8)] is multiplied by the number of APBs determined from the powder diffraction fit. Together with the magnetically dead surface layer this results in a magnetization of 65 (1) A m^2^ per kg_FeOx_, which is slightly larger than the experimental value. A larger affected slab thickness of *t* = 0.55 nm would be needed to achieve the measured magnetization. The presence of multiple APBs may lead to a stronger influence on the spin structure, due to the smaller distance between APBs not allowing for full alignment of moments with increasing distance from the APB. Additionally, in the regions where APBs cross or meet the spin structure, disturbance is likely to be stronger than what is observed for the single APB. Furthermore, accumulation of vacancies and lattice distortions at the APB might further reduce the magnetization. Note also that the determined number of APBs might not reflect the real number but rather gives the number of APBs completely at the centre of the nanoparticle that would lead to the observed peak broadening. It is, however, possible that more APBs than the determined number are present but that they are positioned at a distance from the particle centre. Thus the peak broadening would be the same, but there would be more boundary planes where the spin structure is disordered, which would subsequently result in a stronger reduction of the magnetization. Nevertheless, with the developed description of the APB-induced peak-dependent line broadening, a good fit to the experimental data is obtained and an estimate of the number of APBs can be made. On this basis the lower limit of the reduction in magnetization can be approximated, where the presented values may serve as a reference for future studies.

## 4. Conclusions

In conclusion, to study the influence of antiphase boundaries on the atomic spin structure and X-ray powder diffraction patterns, we performed Monte Carlo and Debye scattering equation simulations for 5, 6.7 and 9.2 nm nanoparticles. Our results suggest that APBs are capable of reducing the observed magnetization of a particle by 3.9% (for 9 nm particles) to 7.9% (for 5 nm particles) in saturation fields of 1.5 T compared with a particle without this defect. This reduction is caused by strong spin disorder at the boundary, while order is restored towards the particle surface, leaving the nanocrystals in a single-domain state. Additionally, we have shown that dipole–dipole interactions have only a small influence on the macroscopic magnetic properties of spherical particles in the size range considered here. Finally, we have presented a method for the detection and quantification of APBs using the powder diffraction method, utilizing the *hkl* dependence of peak broadening. Analysis of experimental data supports the simulation findings and shows that the number of APBs is directly related to the drop in magnetization observed for these particles.

## 5. Supplementary materials: signature of APBs in iron oxide nanoparticles

The simulated structures obtained from the Monte Carlo simulations are given in supplementary text files containing the iron atom positions and the spin vectors.

## Supplementary Material

Additional figures. DOI: 10.1107/S1600576721010128/kc5133sup1.pdf

Simulated structures obtained from the Monte Carlo simulations, given in separate text files containing the iron atom positions and the spin vectors. DOI: 10.1107/S1600576721010128/kc5133sup2.zip

## Appendix A Finite size contribution

The Fourier transform of the particle size component *A*_size_ can be obtained from the intersection volume *V*_*i*_ between a particle of diameter *D* and an identical copy shifted by a distance *L* along the scattering vector normalized to the particle volume *V* according to (Scardi & Leoni, 2004 ▸)

## Appendix B APB contribution

A theory describing the dependence of the peak width on the *hkl* indices was developed by Wilson (1943 ▸) and later corrected by Wilson & Zsoldos (1966 ▸). Warren (1990 ▸) arrived at similar expressions using a slightly different approach (Scardi & Leoni, 2005 ▸). Following Warren, the 1/4*a*_0_[110] APB leads to a phase shift due to the displacement of atoms, whose influence on the diffracted intensity can be expressed as an average phase factor that is given by

where Φ(*n*_1_ *n*_2_ *n*_3_) is the phase shift between a unit cell at the origin and its neighbour at *n*_1_ *n*_2_ *n*_3_. This phase shift is the sum of all phase shifts along the path from the origin at 000 to *n*_1_ *n*_2_ *n*_3_. Thus, the average indicated by angle brackets is the product of the averages in all three real-space directions. The average shift in one direction is the sum of all changes in this direction or, putting it differently, an average shift occurring *n* times, where *n* is the number of possible shifts in that direction. This average shift for pairs of cells  is assumed to be equal for all directions in real space and is the sum of possible phase changes between neighbouring cells times their probability of occurring. For the 1/4*a*_0_[110] APB, the possible atom displacements are along [011], [101] and [110], and the corresponding phase shifts Φ are  ,  and  .

The average phase factors between neighbouring cells along the three real-space directions are

where δ is the probability of a change in phase, *i.e.* crossing an APB, that is assumed to be equal for all directions. The term  corresponds to the probability of no change multiplied by the associated phase factor of 1, *i.e.* no shift. The terms of the form  relate to the phase shifts occurring with probability δ. Thus if Φ = π(2*n*) for all directions, the average phase factor is equal to 1 and no change in the peak widths occurs. If Φ = π(2*n* + 1), a change in phase is observed between the cells and therefore the respective peaks appear broadened. For peak indices with the property *h* + *k* = 4*n*, *h* + *l* = 4*n* and *k* + *l* = 4*n* in all directions, the phase factors evaluate to 1, which is consistent with the observation of unaffected peaks 222, 400 and 440 in the simulations [Fig. 6 ▸(*b*)].

For the peaks that are affected, a distinction can be made on the basis of the peak indices. They can be subdivided into reflections with only even (*a*.1) and only odd indices (*a*.2). For reflections of type *a*.1, equation (13) with equation (14) yields

noting that for this group of reflections one pair of indices will always be divisible by four and, due to the cubic symmetry, the indices can be arranged such that this pair is *h* + *k*. Here again it is assumed that the APB probability is the same in all real-space directions. Within group *a*.2 with only odd indices, there are some peaks with two pairs of indices whose sum is divisible by four. We call this group *a*.2.1 and their average phase factor is

For group *a*.2.2 where all indices are odd and no sum of pairs is divisible by four, the associated phase factor is

The numbers of unit cells traversed by an infinitesimal shift in the direction [*hkl*] can be expressed in terms of the peak indices *h*, *k* and *l* by considering that the number of cells crossed per unit distance is proportional to the angle between two vectors.

In case *a*.1, the sum of the number of cells |*n*_1_| and |*n*_2_| has to be considered, which can be described by the cosine of the angle between [*hkl*] and [110]. To account for the symmetry-equivalent orientations of the lattice planes that contribute to the respective peaks, the cosines of the angles between [*hkl*] and the vectors [*HKL*] ([110], [101], [011],  ,  and  ) are added, giving

With the normalization to the cubic lattice parameter *a*_0_, the APB probability δ obtained from a fit is given as the probability per lattice parameter distance. For *a*.2.1 peaks, only |*n*_1_| is considered and the appropriate exponent is

obtained from [*HKL*] vectors [100], [010] and [001].

Finally, for *a*.2.2 peaks, *e.g.* peaks (511) and (333), the exponent is

where [*HKL*] vectors  ,  ,  and [111] are used.

It can thus be seen that, in general, the Fourier transform of the APB peak profile takes the form

where *L* is the Fourier length (Scardi & Leoni, 2005 ▸). For the different groups of reflections the respective factors *n*(*h*, *k*, *l*) have to be inserted, as given in equations (18), (19) and (20).

## Appendix C Instrumental contribution

The inclusion of the instrumental broadening in equation (9) is straightforward, with the normalized Fourier transform of a pseudo-Voigt function given by

where

Here, the parameter σ controls the peak broadening due to the instrument, and η is a mixing parameter between the Lorentz and Gauss functions to describe the peak shape (Scardi & Leoni, 1999 ▸)

## References

[bb1] Allen, S. M. & Cahn, J. W. (1979). *Acta Metall.* **27**, 1085–1095.

[bb2] Andersen, H. L., Frandsen, B. A., Gunnlaugsson, H. P., Jørgensen, M. R. V., Billinge, S. J. L., Jensen, K. M. Ø. & Christensen, M. (2021). *IUCrJ*, **8**, 33–45. 10.1107/S2052252520013585 PMC7792993 33520241

[bb3] Bailey, R. (1983). *J. Magn. Magn. Mater.* **39**, 178–182.

[bb4] Bedanta, S., Petracic, O. & Kleemann, W. (2015). *Handbook of Magnetic Materials*, Vol. 23, edited by K. H. J. Buschow, pp. 1–83. Amsterdam: Elsevier.

[bb5] Blundell, S. (2001). *Magnetism in Condensed Matter.* Oxford University Press.

[bb6] Bock, N., Riminucci, A., Dionigi, C., Russo, A., Tampieri, A., Landi, E., Goranov, V. A., Marcacci, M. & Dediu, V. (2010). *Acta Biomater.* **6**, 786–796. 10.1016/j.actbio.2009.09.017 19788946

[bb7] Bragg, W. (1940). *Proc. Phys. Soc.* **52**, 105–109.

[bb8] Brown, P. J., Fox, A. G., Maslen, E. N., O’Keefe, M. A. & Willis, B. T. M. (2006). *International Tables for Crystallography*, Vol. C, *Mathematical, Physical and Chemical Tables*, ch. 6.1, pp. 554–595, 1st online ed. Chester: International Union of Crystallography.

[bb9] Cervellino, A., Frison, R., Cernuto, G., Guagliardi, A. & Masciocchi, N. (2014). *J. Appl. Cryst.* **47**, 1755–1761.

[bb10] Coey, J. M. (2010). *Magnetism and Magnetic Materials.* Cambridge University Press.

[bb11] Corot, C., Robert, P., Idée, J.-M. & Port, M. (2006). *Adv. Drug Deliv. Rev.* **58**, 1471–1504. 10.1016/j.addr.2006.09.013 17116343

[bb12] Cromer, D. T. & Mann, J. B. (1968). *Acta Cryst.* A**24**, 321–324.

[bb13] Deatsch, A. E. & Evans, B. A. (2014). *J. Magn. Magn. Mater.* **354**, 163–172.

[bb14] Debye, P. (1915). *Ann. Phys.* **351**, 809–823.

[bb15] Demortiere, A., Panissod, P., Pichon, B., Pourroy, G., Guillon, D., Donnio, B. & Begin-Colin, S. (2011). *Nanoscale*, **3**, 225–232. 10.1039/c0nr00521e 21060937

[bb16] Disch, S., Wetterskog, E., Hermann, R. P., Wiedenmann, A., Vainio, U., Salazar-Alvarez, G., Bergström, L. & Brückel, T. (2012). *New J. Phys.* **14**, 013025.

[bb17] Dobson, J. (2006). *Drug Dev. Res.* **67**, 55–60.

[bb18] Eerenstein, W., Palstra, T. & Hibma, T. (2001). *Thin Solid Films*, **400**, 90–94.

[bb19] Eerenstein, W., Palstra, T., Hibma, T. & Celotto, S. (2002). *Phys. Rev. B*, **66**, 201101. 10.1103/PhysRevLett.88.247204 12059330

[bb20] Eerenstein, W., Palstra, T., Saxena, S. & Hibma, T. (2002). *Phys. Rev. Lett.* **88**, 247204. 10.1103/PhysRevLett.88.247204 12059330

[bb21] Evans, R. F., Fan, W. J., Chureemart, P., Ostler, T. A., Ellis, M. O. & Chantrell, R. W. (2014). *J. Phys. Condens. Matter*, **26**, 103202. 10.1088/0953-8984/26/10/103202 24552692

[bb22] Farrow, C. L. & Billinge, S. J. L. (2009). *Acta Cryst.* A**65**, 232–239. 10.1107/S0108767309009714 19349667

[bb23] Fisher, R. & Marcinkowski, M. (1961). *Philos. Mag.* **6**, 1385–1405.

[bb24] Gilks, D., Lari, L., Naughton, J., Cespedes, O., Cai, Z., Gerber, A., Thompson, S., Ziemer, K. & Lazarov, V. (2013). *J. Phys. Condens. Matter*, **25**, 485004. 10.1088/0953-8984/25/48/485004 24177186

[bb25] Gleich, B. & Weizenecker, J. (2005). *Nature*, **435**, 1214–1217. 10.1038/nature03808 15988521

[bb26] Gonzales-Weimuller, M., Zeisberger, M. & Krishnan, K. M. (2009). *J. Magn. Magn. Mater.* **321**, 1947–1950. 10.1016/j.jmmm.2008.12.017 PMC4578659 26405373

[bb27] Grau-Crespo, R., Al-Baitai, A. Y., Saadoune, I. & De Leeuw, N. H. (2010). *J. Phys. Condens. Matter*, **22**, 255401. 10.1088/0953-8984/22/25/255401 21393797

[bb28] Greaves, C. (1983). *J. Solid State Chem.* **49**, 325–333.

[bb29] Hergt, R., Dutz, S., Müller, R. & Zeisberger, M. (2006). *J. Phys. Condens. Matter*, **18**, S2919–S2934.

[bb30] Herlitschke, M., Disch, S., Sergueev, I., Schlage, K., Wetterskog, E., Bergström, L. & Hermann, R. P. (2016). *J. Phys. Conf. Ser.* **711**, 012002.

[bb31] Herynek, V., Babič, M., Kaman, O., Charvátová, H., Veselá, M., Buchholz, O., Vosmanská, M., Kubániová, D., Kohout, J., Hofmann, U. G. & Šefc, L. (2021). *J. Nanopart. Res.* **23**, 1–15.

[bb32] Hibma, T., Voogt, F., Niesen, L., van der Heijden, P., de Jonge, W., Donkers, J. & van der Zaag, P. (1999). *J. Appl. Phys.* **85**, 5291–5293.

[bb33] Johannsen, M., Gneveckow, U., Eckelt, L., Feussner, A., Waldöfner, N., Scholz, R., Deger, S., Wust, P., Loening, S. & Jordan, A. (2005). *Int. J. Hyperthermia*, **21**, 637–647. 10.1080/02656730500158360 16304715

[bb34] Jørgensen, J.-E., Mosegaard, L., Thomsen, L. E., Jensen, T. R. & Hanson, J. C. (2007). *J. Solid State Chem.* **180**, 180–185.

[bb35] Josephson, L., Tung, C.-H., Moore, A. & Weissleder, R. (1999). *Bioconjugate Chem.* **10**, 186–191. 10.1021/bc980125h 10077466

[bb36] Jun, Y.-W., Lee, J.-H. & Cheon, J. (2008). *Angew. Chem. Int. Ed.* **47**, 5122–5135. 10.1002/anie.200701674 18574805

[bb37] Kikuchi, R. & Cahn, J. W. (1979). *Acta Metall.* **27**, 1337–1353.

[bb38] Kodama, R. H. & Berkowitz, A. E. (1999). *Phys. Rev. B*, **59**, 6321–6336.

[bb39] Köhler, T., Feoktystov, A., Petracic, O., Kentzinger, E., Bhatnagar-Schöffmann, T., Feygenson, M., Nandakumaran, N., Landers, J., Wende, H., Cervellino, A., Rücker, U., Dunin-Borkowski, R. & Brückel, T. (2021). *Nanoscale*, **13**, 6965–6976. 10.1039/d0nr08615k 33885498

[bb40] Krishnan, K. M. (2010). *IEEE Trans. Magn.* **46**, 2523–2558. 10.1109/TMAG.2010.2046907 PMC2949969 20930943

[bb41] Kumar, C. S. & Mohammad, F. (2011). *Adv. Drug Deliv. Rev.* **63**, 789–808. 10.1016/j.addr.2011.03.008 PMC3138885 21447363

[bb42] Laurent, S., Dutz, S., Häfeli, U. O. & Mahmoudi, M. (2011). *Adv. Colloid Interface Sci.* **166**, 8–23. 10.1016/j.cis.2011.04.003 21601820

[bb43] Laurent, S., Forge, D., Port, M., Roch, A., Robic, C., Vander Elst, L. & Muller, R. N. (2008). *Chem. Rev.* **108**, 2064–2110. 10.1021/cr068445e 18543879

[bb44] Lee, S.-J. & Lee, S. (2006). *New J. Phys.* **8**, 98.

[bb45] Levy, M., Quarta, A., Espinosa, A., Figuerola, A., Wilhelm, C., García-Hernández, M., Genovese, A., Falqui, A., Alloyeau, D., Buonsanti, R., Cozzoli, P. D., García, M. A., Gazeau, F. & Pellegrino, T. (2011). *Chem. Mater.* **23**, 4170–4180.

[bb46] Luysberg, M., Sofin, R., Arora, S. & Shvets, I. (2009). *Phys. Rev. B*, **80**, 024111.

[bb47] Ma, X., Gong, A., Chen, B., Zheng, J., Chen, T., Shen, Z. & Wu, A. (2015). *Colloids Surf. B Biointerfaces*, **126**, 44–49. 10.1016/j.colsurfb.2014.11.045 25543982

[bb48] McKenna, K. P., Hofer, F., Gilks, D., Lazarov, V. K., Chen, C., Wang, Z. & Ikuhara, Y. (2014). *Nat. Commun.* **5**, 1–8. 10.1038/ncomms6740 PMC4275585 25494005

[bb49] Momma, K. & Izumi, F. (2011). *J. Appl. Cryst.* **44**, 1272–1276.

[bb50] Moreno, R., Jenkins, S., Skeparovski, A., Nedelkoski, Z., Gerber, A., Lazarov, V. K. & Evans, R. F. L. (2021). *J. Phys. Condens. Matter*, **33**, 175802. 10.1088/1361-648X/abe26c 33530069

[bb51] Nakajima, K., Hirotsu, Y. & Okamoto, S. (1987). *J. Am. Ceram. Soc.* **70**, 321–322.

[bb52] Nedelkoski, Z., Kepaptsoglou, D., Lari, L., Wen, T., Booth, R. A., Oberdick, S. D., Galindo, P. L., Ramasse, Q. M., Evans, R. F., Majetich, S. & Lazarov, V. K. (2017). *Sci. Rep.* **7**, 45997. 10.1038/srep45997 PMC5385549 28393876

[bb53] Nemati, Z., Alonso, J., Rodrigo, I., Das, R., Garaio, E., García, J. Á., Orue, I., Phan, M.-H. & Srikanth, H. (2018). *J. Phys. Chem. C*, **122**, 2367–2381.

[bb54] Newman, M. & Barkema, G. (1999). *Monte Carlo Methods in Statistical Physics.* Oxford University Press.

[bb55] Pareta, R. A., Taylor, E. & Webster, T. J. (2008). *Nanotechnology*, **19**, 265101. 10.1088/0957-4484/19/26/265101 21828670

[bb56] Raj, K., Moskowitz, B. & Casciari, R. (1995). *J. Magn. Magn. Mater.* **149**, 174–180.

[bb57] Ramachandran, P. & Varoquaux, G. (2011). *Comput. Sci. Eng.* **13**, 40–51.

[bb58] Rudolph, M., Motylenko, M. & Rafaja, D. (2019). *IUCrJ*, **6**, 116–127. 10.1107/S2052252518015786 PMC6327185 30713709

[bb59] Scardi, P. & Leoni, M. (1999). *J. Appl. Cryst.* **32**, 671–682.

[bb60] Scardi, P. & Leoni, M. (2004). *Diffraction Analysis of the Microstructure of Matererials*, pp. 51–91. Heidelberg: Springer.

[bb61] Scardi, P. & Leoni, M. (2005). *Acta Mater.* **53**, 5229–5239.

[bb62] Scardi, P., Ortolani, M. & Leoni, M. (2010). *Mater. Sci. Forum*, **651**, 155–171.

[bb63] Semelka, R. C. & Helmberger, T. K. (2001). *Radiology*, **218**, 27–38. 10.1148/radiology.218.1.r01ja2427 11152776

[bb64] Sharifi Dehsari, H., Ksenofontov, V., Möller, A., Jakob, G. & Asadi, K. (2018). *J. Phys. Chem. C*, **122**, 28292–28301.

[bb65] Shmakov, A. N., Kryukova, G. N., Tsybulya, S. V., Chuvilin, A. L. & Solovyeva, L. P. (1995). *J. Appl. Cryst.* **28**, 141–145.

[bb66] Sun, C., Lee, J. S. & Zhang, M. (2008). *Adv. Drug Deliv. Rev.* **60**, 1252–1265. 10.1016/j.addr.2008.03.018 PMC2702670 18558452

[bb67] Sun, Y.-P. (2002). *Supercritical Fluid Technology in Materials Science and Engineering: Syntheses, Properties, and Applications.* Boca Raton: CRC Press.

[bb68] Tran, N. & Webster, T. J. (2009). *WIREs Nanomed Nanobiotechnol*, **1**, 336–351. 10.1002/wnan.23 20049801

[bb69] Veiseh, O., Gunn, J. W. & Zhang, M. (2010). *Adv. Drug Deliv. Rev.* **62**, 284–304. 10.1016/j.addr.2009.11.002 PMC2827645 19909778

[bb70] Warren, B. E. (1990). *X-ray Diffraction.* North Chelmsford: Courier Corporation.

[bb71] Wetterskog, E., Tai, C.-W., Grins, J., Bergström, L. & Salazar-Alvarez, G. (2013). *ACS Nano*, **7**, 7132–7144. 10.1021/nn402487q 23899269

[bb72] Willmott, P. R., Meister, D., Leake, S. J., Lange, M., Bergamaschi, A., Böge, M., Calvi, M., Cancellieri, C., Casati, N., Cervellino, A., Chen, Q., David, C., Flechsig, U., Gozzo, F., Henrich, B., Jäggi-Spielmann, S., Jakob, B., Kalichava, I., Karvinen, P., Krempasky, J., Lüdeke, A., Lüscher, R., Maag, S., Quitmann, C., Reinle-Schmitt, M. L., Schmidt, T., Schmitt, B., Streun, A., Vartiainen, I., Vitins, M., Wang, X. & Wullschleger, R. (2013). *J. Synchrotron Rad.* **20**, 667–682. 10.1107/S0909049513018475 PMC3747948 23955029

[bb73] Wilson, A. J. C. (1943). *Proc. Math. Phys. Eng. Sci.* **181**, 360–368.

[bb74] Wilson, A. J. C. & Zsoldos, L. (1966). *Proc. Math. Phys. Eng. Sci.* **290**, 508–514.

