# Supplementary material for: Signature of antiphase boundaries in iron oxide nanoparticles
Source: J Appl Crystallogr. 2021 Nov 16;54(Pt 6):1719–29. doi: 10.1107/S1600576721010128 (PMC8662974; doi:10.1107/S1600576721010128)
Supplement: Supplementary file 1 [file j-54-01719-sup1.pdf]

JOURNAL OF APPLIED CRYSTALLOGRAPHY

Volume 54 (2021)

Supporting information for article:

Signature of antiphase boundaries in iron oxide nanoparticles

Tobias Köhler, Artem Feoktystov, Oleg Petracic, Nileena Nandakumaran, Antonio Cervellino and Thomas Brückel

Supplementary materials: Signature of antiphase boundaries in iron oxide nanoparticles

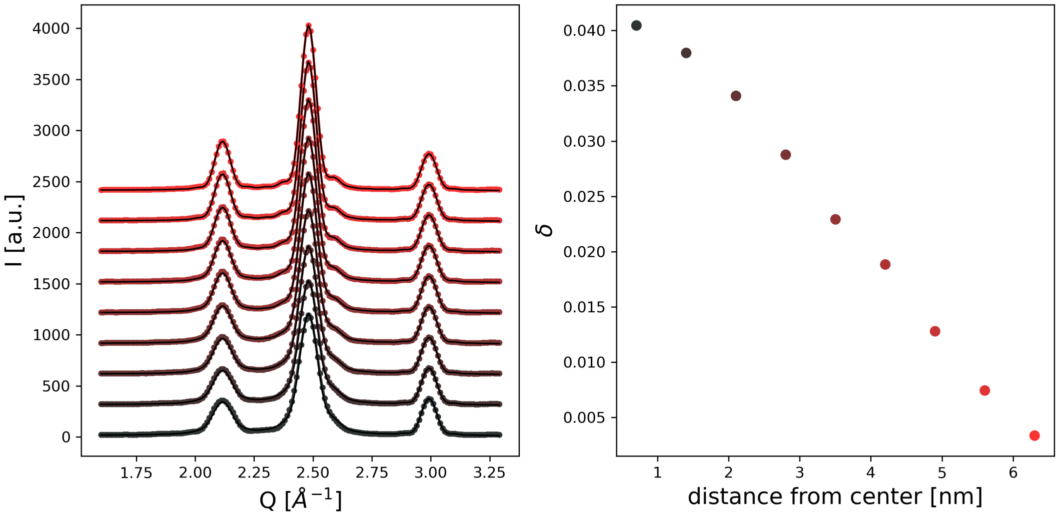

Fig. S1. Simulation of powder diffraction patterns for different placements of the APB within a particle of 11 unit cells in diameter. The peak broadening depends on the placement and is strongest for an APB through the particle center. This is also reflected in the APB probability parameter δ determined from fits to the simulated patterns, as shown on the right hand side. The decrease in δ with increasing distance from the particle center is related to the decrease of the relative area of the APB to the sphere cross section.

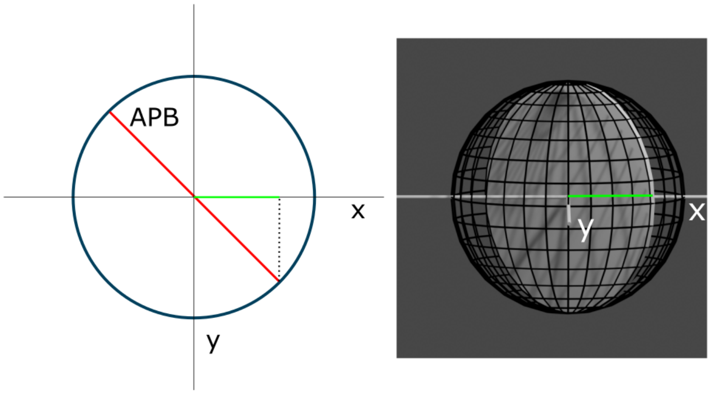

Fig. S2. Sketch of the APB in a spherical particle. The ratio between the ellipse area, corresponding to the area of the APB viewed along x or y, to the sphere cross section leads to the factor $\sqrt{2}$ in eq. 11.

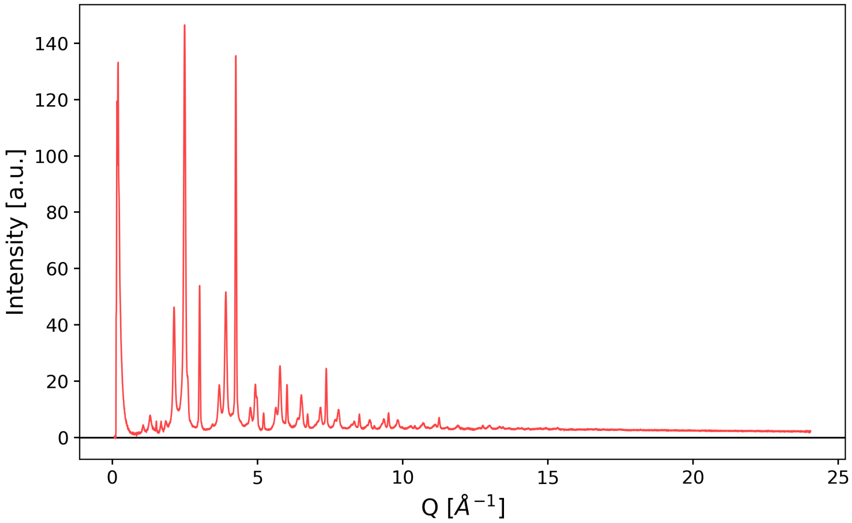

Fig. S3. Complete X-ray powder diffraction data obtained from a dried sample of nanoparticles. A smaller region of this dataset was used for fig. 7.

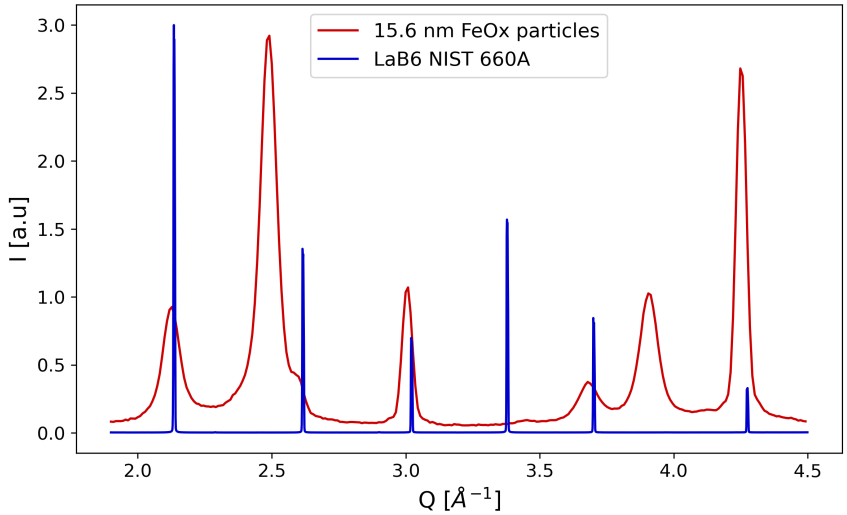

Fig. S4. Comparison of X-ray powder diffraction patterns for the iron oxide nanoparticle sample used in this work and for the NIST standard sample LaB_6 recorded under the same conditions at the beamline MS-X04SA of the SLS. A wavelength of 0.4329 Å was used.

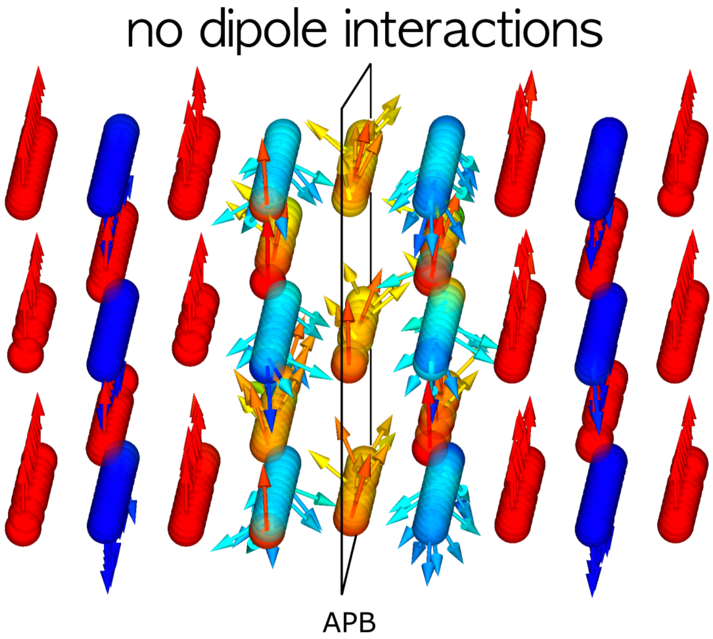

Fig. S5. Enlarged part of the spin structures of the 9.2 nm particles with an APB in the center, indicated with the black rectangle. The structures were simulated without consideration of dipole-dipole interactions (visualized with MayaVi (Ramachandran & Varoquaux, 2011)). The field of 5 T was applied along the vertical direction. Full parallel (octahedral sites) and antiparallel (tetrahedral sites) alignment with the applied field is shown with red and blue colors, respectively. Intermediate colors indicate spin canting away from the perfect alignment. Introduction of an APB, leads to a disturbance in the spin structure due to the antiferromagnetic exchange interaction across the boundary.

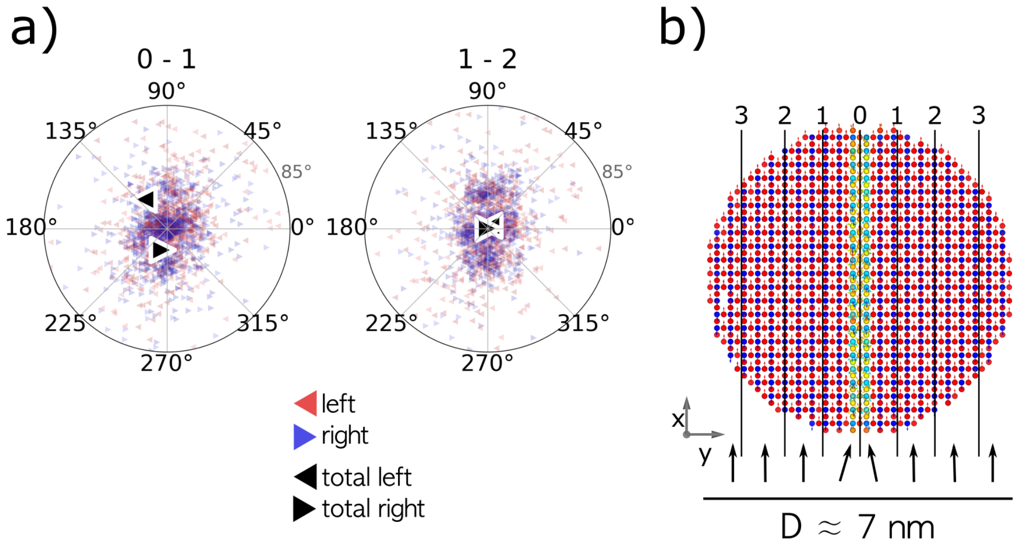

Fig. S6. a) Net magnetization vectors for slices to the left and to the right hand side of the APB (large black triangles) together with the individual spin vectors (small triangles) for 6.7 nm particles, simulated with consideration of dipole-dipole interactions. The slab positions are indicated at the top with numbers relating to the slices shown in b). The canting of spins is confined to the region within one unit cell around the APB. This is indicated in an exaggerated way by the arrows below the particle shown in b).

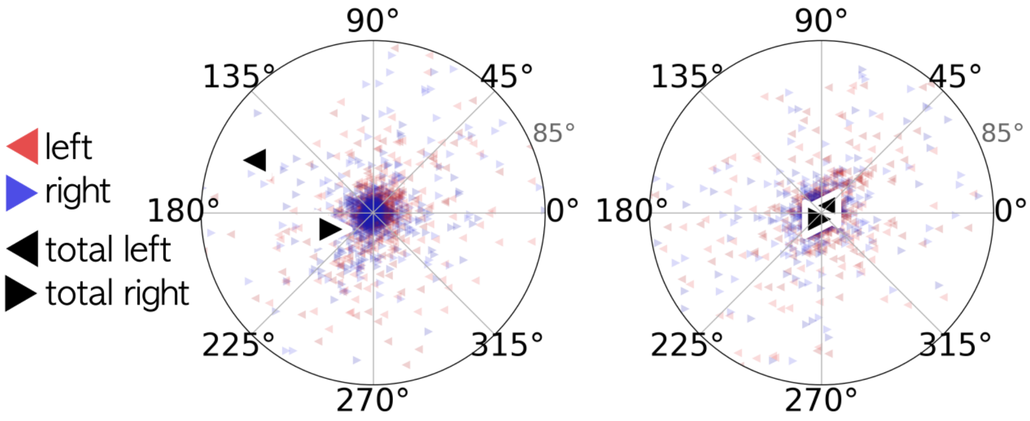

Fig. S7. Net magnetization vectors for slices to the left and to the right hand side of the APB (large black triangles) together with the individual spin vectors (small triangles) for 6.7 nm particles simulated without consideration of dipole-dipole interactions. The slab positions are indicated at the top with numbers relating to the slices shown in fig. S6b. The canting of spins is confined to the region within one unit cell around the APB.

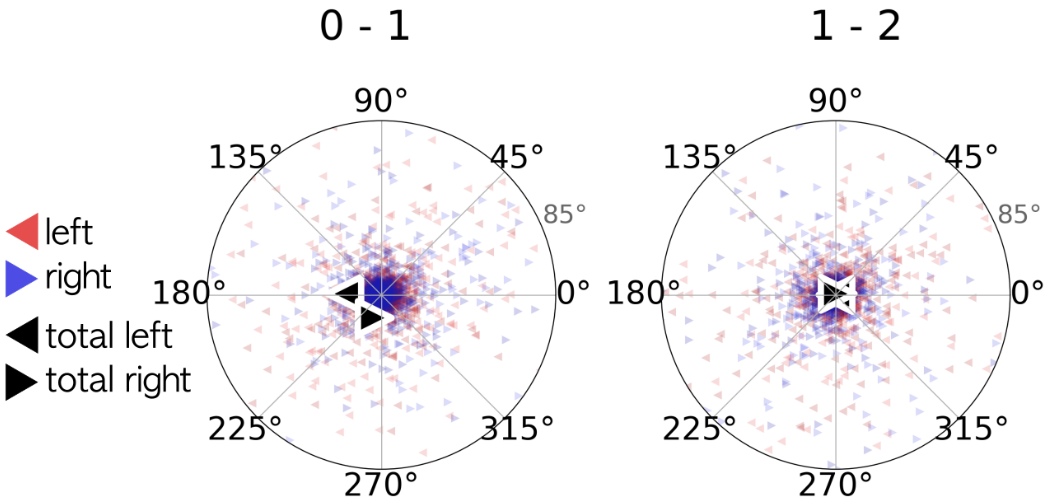

Fig. S8. Net magnetization vectors for slices to the left and to the right hand side of the APB (large black triangles) together with the individual spin vectors (small triangles) for 9.2 nm particles simulated without consideration of dipole-dipole interactions. The slab positions are indicated at the top with numbers relating to the slices shown in fig. 5b. The canting of spins is confined to the region within one unit cell around the APB.

Simulated structures

The simulated structures obtained from the Monte Carlo simulations are given in separate text files containing the iron atom positions and the spin vectors.

1. Spin_structure_7nm_5T_APB.txt
2. Spin_structure_7nm_5T_noAPB.txt
3. Spin_structure_9nm_5T_APB.txt
4. Spin_structure_9nm_5T_noAPB.txt
